# Estrogen receptor α as a predictive biomarker for survival in human papillomavirus-positive oropharyngeal squamous cell carcinoma

**DOI:** 10.1186/s12967-020-02396-8

**Published:** 2020-06-16

**Authors:** Soohyeon Kwon, Soon-Hyun Ahn, Woo-Jin Jeong, Young Ho Jung, Yun Jung Bae, Jin Ho Paik, Jin-Haeng Chung, Hyojin Kim

**Affiliations:** 1 Department of Pathology, Seoul National University Bundang Hospital, Gumi-ro 173-Beon-gil 82, Bundang-gu, Seongnam, Gyeonggi-do 13620 Republic of Korea; 2 Department of Otorhinolaryngology, Seoul National University Hospital, Seoul, Republic of Korea; 3 Department of Otorhinolaryngology, Seoul National University Bundang Hospital, Seongnam, Republic of Korea; 4 Department of Radiology, Seoul National University Bundang Hospital, Seongnam, Republic of Korea

**Keywords:** Estrogen receptor α, Oropharynx, Squamous cell carcinoma, Human papillomavirus, Biomarker, Prognosis

## Abstract

**Background:**

Although oropharyngeal squamous cell carcinoma (OPSCC) with human papillomavirus (HPV) infection has a good prognosis, the accurate prediction of survival and risk of treatment failure is essential to design deintensification regimens. Here, we investigated estrogen receptor α (ERα) as a prognostic biomarker with therapeutic implications in OPSCC alongside factors associated with HPV infection.

**Methods:**

We performed immunohistochemistry for ERα and p53 using formalin-fixed, paraffin-embedded tissues and assessed the HPV status using p16 immunohistochemistry and HPV DNA testing in 113 consecutive patients with OPSCC treated with surgical resection or radiotherapy/chemoradiotherapy.

**Results:**

ERα expression and p53 alteration was observed in 35.4% and 21.2% OPSCCs; 45.6% and 1.3% p16+/HPV+ OPSCCs; and 11.5% and 76.9% p16− OPSCCs, respectively. These data suggest that OPSCC pathogenesis varies with HPV status. Furthermore, ERα expression was associated with improved overall survival (OS) in both HPV+ (p16+/HPV+ OPSCC) and p16+ (p16+ OPSCC irrespective of HPV status) models (*p* = 0.005 and *p* = 0.006, respectively) and with improved OS adjusted for stage (*p* = 0.037, hazard ratio: 0.109, 95% confidence interval 0.013–0.871) in the p16+ model.

**Conclusions:**

ERα is a potential predictive biomarker for improved survival in both HPV+ and p16+ OPSCC models.

## Background

Two main causes of oropharyngeal squamous cell carcinoma (OPSCC) are human papillomavirus (HPV) infection and tobacco and alcohol abuse, and the resulting OPSCCs are referred to as HPV-positive (HPV+) and HPV-negative (HPV−) OPSCCs, respectively [1–3]. The incidence of OPSCC in developed countries is increasing continuously and is ~ 70–80%, mainly because of increasing HPV infection [4, 5]. The clinical characteristics and outcomes in patients with HPV+ OPSCC are significantly different from those in patients with HPV− OPSCC [6, 7]. Therefore, the American Joint Committee on Cancer (AJCC) and the Union for International Cancer Control developed a distinct staging algorithm specific to HPV+ OPSCC in their staging guidelines (8th edition) [8, 9]. Since the adoption of the 8th edition AJCC guidelines, several deintensification trials were conducted to investigate the feasibility of omitting concomitant chemotherapy in the definitive or adjuvant radiotherapy (RT) settings owing to the good prognosis of HPV+ OPSCC and the adverse effects of systemic therapy [10–14]. Unfortunately two large-scale phase III trials, RTOG 1016 and ESCALaT, that replaced concurrent chemoradiotherapy (CRT) with cetuximab + chemotherapy showed poor survival, and systemic therapy was found to improve the clinical outcomes for some patients with 8th edition AJCC-based stage I HPV+ OPSCC [10, 13]. Consequently, there is an urgent need for prognostic biomarkers and guidelines for treatment deintensification in HPV+ OPSCC.

Estrogen receptors (ERs) exist in two isoforms, ERα and ERβ. These isoforms trigger distinct transcriptional responses and exert opposite effects on cellular processes, including proliferation, apoptosis, migration, and other processes that differentially influence cancer development and progression [15]. Although the role of ERβ in cancer biology remains controversial, ERα is well known as an important factor involved in tumorigenesis and cancer progression [16–18]. The Cancer Genome Atlas data analysis of an OPSCC cohort revealed the highest ERα mRNA expression in patients with HPV+ OPSCC, and patients with ERα protein expression showed improved survival after adjusting for clinical risk factors including HPV status [19]. Furthermore, ERα was significantly associated with improved overall survival (OS) in patients with HPV+ OPSCC [20]. This prognostic implication of ERα in HPV+ OPSCC is considerably different from the known role of ERα in HPV+ cervical cancer.

We focused on whether ERα expression affects the new staging system in predicting in patients with OPSCC. We would like to assess the possibility of using ERα expression to design a variety of treatment options within the same step in a clinical setting. Therefore, we investigated the ERα expression in OPSCC under the 8th edition AJCC staging system with respect to the p16/HPV status and explored the prognostic effect of ERα expression, especially in HPV+ OPSCC.

## Methods

### Study population

We included 113 patients with biopsy-confirmed, loco-regionally confined OPSCC treated with curative intent, surgical resection, or RT/CRT at Seoul National University Bundang Hospital between January 2004 and January 2013. We excluded the patients undergoing palliative treatment, patients currently undergoing or previously treated for other squamous cell carcinoma (SCC) in the head and neck region, and patients with histology other than SCC or subtype of SCC.

We collected the following clinicopathological data of the patients: age, sex, tobacco use, tumor subsite, primary treatment, tumor recurrence, and status at last follow-up. Initial and pathologic stages according to 7th edition AJCC staging system were determined and retrospectively re-evaluated per the 8th edition AJCC staging system [9]. The study protocol was approved by the Institutional Review Board of Seoul National University Bundang Hospital and informed consent was waived (IRB No. B-2001-589-103).

Of the 113 patients, 68 (60.2%) underwent primary surgery, 45 of whom received postoperative adjuvant RT or CRT. The remaining 45 of 113 patients (39.8%) received definitive oncological treatment (RT or CRT), 21 of whom underwent complementary surgery after neoadjuvant treatment.

### Tumor samples and tissue microarray (TMA) construction

Formalin-fixed paraffin-embedded blocks from biopsy specimens (n = 33) or resected specimens (n = 80) were used for the analyses. TMAs were constructed for resected specimens. In brief, representative core tissue sections (diameter: 4 mm) were excised from individual OPSCC paraffin blocks (donor blocks) and arranged in new TMA blocks using a trephine apparatus (SuperBioChips Laboratories, Seoul, Korea). To minimize the effect of protein expression heterogeneity, three cores were sampled and included in the TMA block from each patient.

### HPV DNA genotyping and p16 immunostaining

HPV status was determined by HPV genotyping and p16 immunohistochemistry (IHC) using the complete resected section and biopsy specimens. HPV genotyping was performed using peptic nucleic acid probe-based fluorescence melting curve analysis in a real-time PCR system (PANA RealTyper™ HPV Kit, PANAGENE, Daejeon, Republic of Korea) according to the manufacturer’s instructions and as described in Additional file 1.

P16 IHC (clone E6H4, CINtec^®^, Ventana Medical Systems, Inc., Tucson, AZ, USA) was performed on an automated platform (Benchmark Ultra; Ventana Medical Systems) according to the manufacturer’s instructions. A positive test was defined as diffuse (> 75%) tumor expression with at least moderate-intensity staining, localized to both the cytoplasm and the nucleus [9]. Owing to the prognostic relevance of HPV DNA status in p16+ OPSCC, the patients were divided into three groups based on p16 and HPV: p16+/HPV+; p16+/HPV−; and p16−/HPV±.

### Immunostaining and interpretation of ERα and p53

IHC was performed on the TMA sections (4 μm) using the Benchmark Ultra automated staining system for ERα and p53. Immunostaining was performed using monoclonal rabbit anti-human ERα (clone SP1, ready-to-use; Ventana Medical Systems) and monoclonal mouse anti-human p53 (clone DO-7, 1:1000, Dako, Carpinteria, CA, USA) primary antibodies. The results were independently interpreted by two pathologists (S.K. and H.K.).

ERα expression was scored using a modified Allred score; the samples were considered ERα-positive if more than 1% cancer cells showed nuclear staining, per the American Society of Clinical Oncology/College of American Pathologists guidelines for breast cancer [21, 22]. Known ERα-positive breast cancer and endometrial specimens were used as positive controls. p53 expression was classified as diffuse strong nuclear staining in > 60% of tumor, complete absence of staining, and focal mild–moderate nuclear staining [23, 24]. The first two patterns are altered expressions that reflect missense or silent mutations in the *p53* gene, and the last one is classified as wild type.

### RNA in situ hybridization of *ESR1* mRNA

*ESR1* mRNAs were measured using RNAscope^®^ assays (Advanced Cell Diagnostics [ACD], Hayward, CA, USA) following the manufacturer’s instructions [25]. Briefly, 4-μm-thick sections were deparaffinized; incubated with pretreatment reagents 1, 2, and 3 at room temperature for 10 min; boiled for 15 min; and incubated at 40 °C for 30 min. Tissue sections were then hybridized with Hs-ESR1-probes (ACD) at 40 °C for 2 h. Hybridization signals were amplified and visualized with an RNAscope^®^ 2.5 HD-Brown Reagent Kit. RNAscope^®^ results were examined under a standard bright field microscope at 400× magnification. Positive signals presented as brown punctuate dots. PPIB and DapB were used as positive and negative probes, respectively, to control tissue RNA conditions and nonspecific hybridization. *ESR1* mRNA signals were in the tumor compartment, as visualized by brown dotted or clustered patterns. We adopted the RNAscope^®^ system scoring guidelines (“RNA scope score”): 0 (no staining or < 1 dot per 10 cells); 1 (1–3 dots per cell); 2 (4–9 dots per cell); 3 (10–15 dots per cell); and 4 (> 15 dots per cell and > 10% dots in clusters) [25], and cases showing RNA scope^®^ score of 1 or more were designated as *ESR1* mRNA positive.

### Statistical analysis

We used SPSS version 25.0 (SPSS Inc., Chicago, IL, USA) to analyze all the data. Chi-squared test and logistic regression were performed to compare assays and determine appropriate cut-off values. Cohen’s coefficient of agreement was obtained to validate the results. Kaplan–Meier analysis was performed to construct survival curves, and statistical significance was assessed using log-rank tests. Multivariate analysis was performed using the Cox proportional hazards regression model. All statistical tests were two sided, and *p* values < 0.05 were considered to indicate statistical significance.

## Results

### Clinicopathologic characteristics

The clinicopathologic features of the patients are summarized in Table 1. Compared to the p16− OPSCC group, the p16+/HPV+ OPSCC group showed higher number of individuals under 65 years of age and never smokers. Most p16+/HPV+ tumors occurred in the palatine tonsil and base of tongue, but p16− tumors occurred in various subsites such as pharyngeal walls, soft palates, and uvula, thereby showing significant differences in tumor origin (*p* < 0.001). Patients with p16+/HPV− and p16+/HPV+ OPSCC share similar baseline characteristics, including age, smoking history, and tumor subsite. Compared to the p16− subgroup, the p16+/HPV+ and p16+/HPV− subgroups showed lower stages per the 8th edition AJCC staging systems (*p *< 0.001).

**Table 1:** Clinicopathologic characteristics

Characteristics	All patients	p16+/HPV+ group	p16+/HPV− group	p16−/HPV± group	*p* value
Sex					
Male	101 (89.4%)	69 (87.3%)	7 (87.5%)	25 (96.2%)	0.442
Female	12 (10.6%)	10 (12.7%)	1 (12.5%)	1 (3.8%)	
Age (years)					
< 65	73 (64.6%)	55 (69.6%)	5 (62.5%)	13 (50%)	0.191
≥ 65	40 (35.4%)	24 (20.4%)	3 (37.5%)	13 (50%)	
Smoking history					
Never	38 (33.6%)	30 (38.0%)	3 (37.5%)	5 (19.2%)	0.083
Ever	75 (66.4%)	49 (62.0%)	5 (62.5%)	21 (80.8%)	
Subsite					
Palatine tonsil	85 (75.2%)	68 (86.1%)	6 (75%)	11 (42.3%)	< 0.001*
Base of tongue	14 (12.4%)	7 (8.9%)	2 (25%)	5 (19.2%)	
Pharyngeal wall	7 (6.2%)	4 (5.1%)	0	3 (11.5%)	
Soft palate	5 (4.4%)	0	0	5 (19.2%)	
Uvula	2 (1.8%)	0	0	2 (7.7%)	
Surgical margin^a^					
Clear	64 (80%)	39 (72.2%)	5 (83.3%)	20 (100%)	0.084
Involved	16 (20%)	15 (27.8%)	1 (16.7%)	0	
Lymphovascular invasion^a^					
Absent	48 (60%)	30 (55.6%)	3 (50%)	15 (75%)	0.478
Present	32 (40%)	24 (44.4%)	3 (50%)	5 (25%)	
Perineural invasion^a^					
Absent	74 (92.5%)	50 (92.6%)	5 (83.3%)	19 (95%)	0.784
Present	6 (7.5%)	4 (7.4%)	1 (16.7%)	1 (5%)	
Initial stage (8th AJCC)					
I	54 (47.8%)	46 (58.2%)	3 (37.5%)	5 (19.2%)	< 0.001*
II	36 (31.9%)	27 (34.2%)	5 (62.5%)	5 (19.2%)	
III	10 (8.8%)	6 (7.6%)	0	4 (15.4%)	
IV	13 (11.5%)	0	0	12 (46.2%)	
ERα					
Positive	40 (35.4%)	36 (45.6%)	1 (12.5%)	3 (11.5%)	0.003*
Negative	73 (64.6%)	43 (54.4%)	7 (87.5%)	23 (88.5%)	
*ESR1* mRNA^b^					
Positive	16 (15.8%)	15 (21.1%)	0	1 (4.3%)	0.079
Negative	85 (84.2%)	56 (78.9%)	7 (100%)	22 (95.7%)	
p53 expression					
Altered	24 (21.2%)	1 (1.3%)	3 (37.5%)	20 (76.9%)	< 0.001*
Wild type	89 (78.8%)	78 (98.7%)	5 (62.5%)	6 (23.1%)	
Total	113 (100%)	79 (69.9%)	8 (7.1%)	26 (23%)	

*HPV* human papillomavirus, *AJCC* American Joint Committee on Cancer, *ERα* estrogen receptor, *ESR1* estrogen receptor 1

*p < 0.05

^a^Evaluated only in 80 surgical resection specimens

^b^Evaluated only in 101 specimens due to RNA quality

### Expression of ERα protein and *ESR1* mRNA in OPSCC

**Fig. 1:**
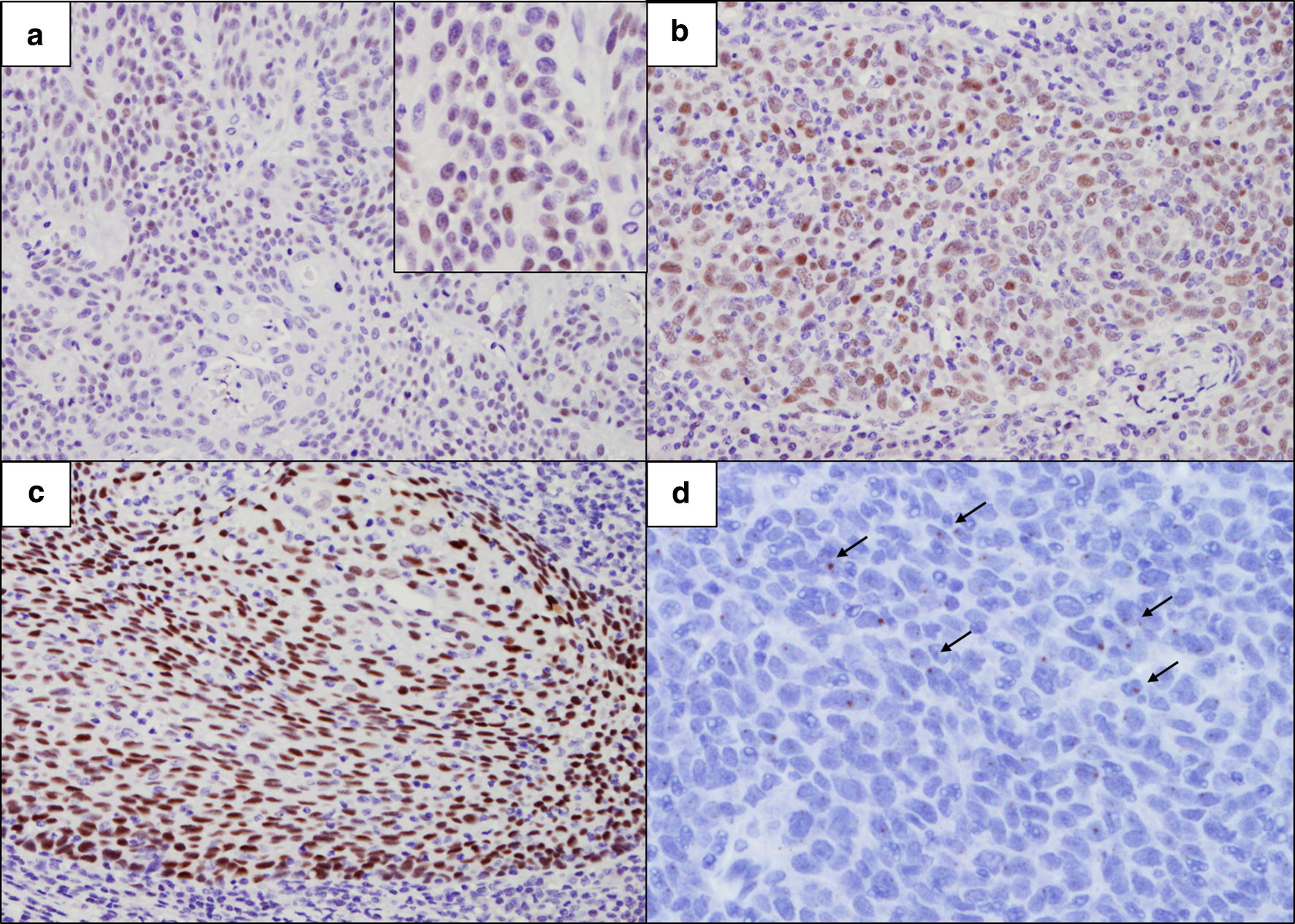
ERα protein (**a**–**c**) and *ESR1* mRNA (**d**) expression in oropharyngeal squamous cell carcinoma. Weak to moderate ERα expression < 10% of tumor cells (**a** ×400 magnification). Weak to moderate ERα expression in tumor cells diffusely (10–90%) (**b** ×400 magnification). Strong ERα expression in tumor cells diffusely (≥ 75%) (**c** ×400 magnification). *ESR1* mRNA expression visualized by brown dotted (arrow) in tumor compartment (**d** ×600 magnification)

**Fig. 2:**
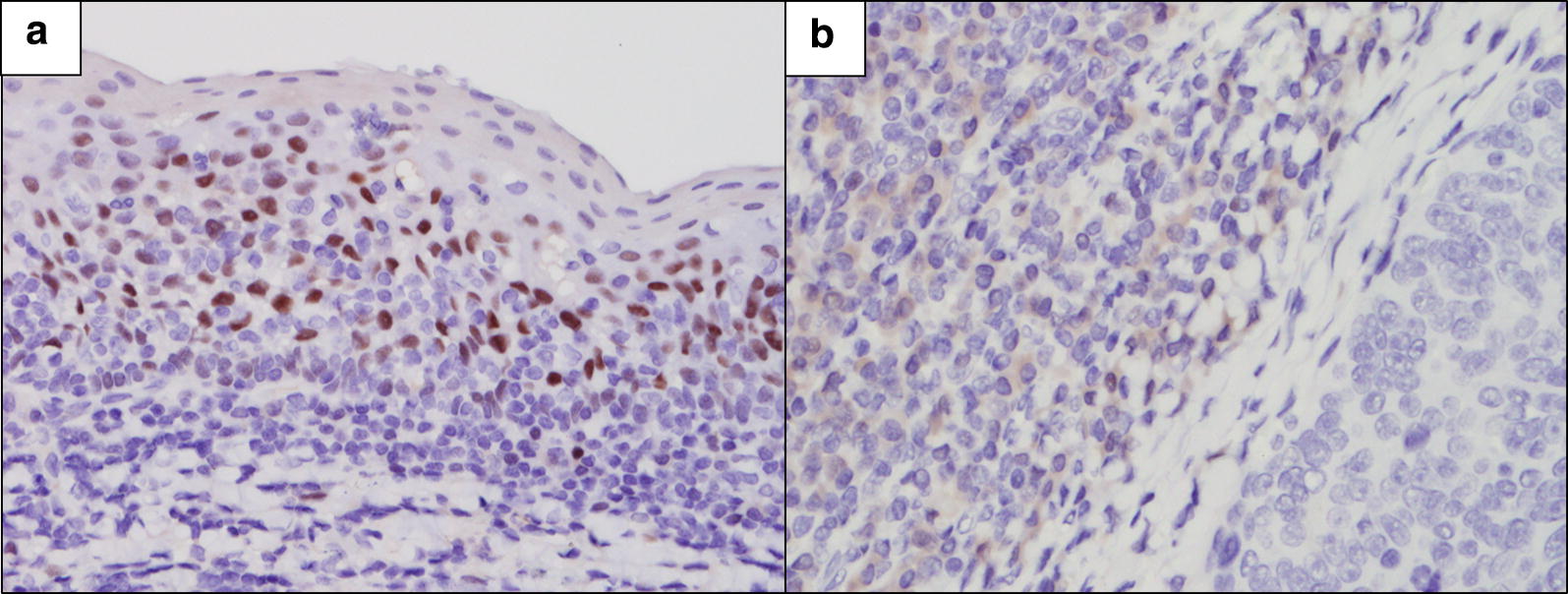
ERα expression in adjacent normal tissue (×400 magnification). ERα was expressed in patches in the basal layer of the non-neoplastic squamous epithelium around the tumor, even in HPV− OPSCC (**a**). ERα expression was not observed in stromal cell nucleus and was weak in lymphocyte cytoplasm (**b**)

One-third of the OPSCCs (35.4%, 40/113) expressed the ERα protein. The intensity of ERα protein showed a linear correlation with the percentage of stained area (r = 0.68, *p* < 0.001). We combined the two criteria and divided the ERα expression pattern into four groups; focal (< 10%) weak to moderate (n = 11, 27.5%), diffuse (≥ 10%) weak to moderate (n = 24, 60%), focal strong (n = 0), and diffuse strong (n = 5, 12.5%) (Fig. 1a–c). ERα expression was restricted to the subsets of basal cells of the non-neoplastic squamous epithelium around the tumor, and this expression was present irrespective of the HPV status of the tumor in 15 out of 80 resected specimens (Fig. 2a). Although ERα expression was not observed in the nuclei of stromal cells, weak staining was observed in the cytoplasm of lymphocytes (Fig. 2b).

*ESR1* mRNA was evaluated in 101 cases except for the 12 cases with poor RNA quality. *ESR1* mRNA expression was observed in 16 (15.8%), and all cases showed ERα protein expression diffusely (Additional file 2). *ESR1* mRNA was expressed at a low level of RNA scope score 1 (1–3 dot per cell) in all cases regardless of the ER protein expression pattern (Fig. 1d).

### ERα, *ESR1* and p53 expression in OPSCC with different p16/HPV status

ERα was more frequently expressed in the p16+/HPV+ subgroup (36/79, 45.6%) than in the p16− subgroup (3/26, 11.5%; *p* = 0.003; Table 1). The expression of *ESR1* mRNA was also higher in the p16+/HPV+ subgroup than p16− subgroup (21.1% vs. 4.3%), which was similar to ERα but there was no statistical significance (*p* = 0.079). Conversely, p53 expression was altered only in one patient in the p16+/HPV+ subgroup (1.3%), but in 76.9% (20/26) patients in the p16− subgroup (*p* < 0.001), suggesting that OPSCC pathogenesis differs with the HPV status. In the p16+/HPV− subgroup, ERα and altered p53 expression was observed in 13.5% (1/8) and 37.5% (3/8) patients, respectively, similar to that observed in the p16− subgroup; however, the number of samples is limited.

### Clinicopathological analysis with respect to ERα and *ESR1* expression in the p16+/HPV+ OPSCC subgroup

We analyzed the differences in the clinicopathologic variables with respect to the ERα expression in the p16+/HPV+ subgroup (Table 2). The tumor stage was lower in ERα-positive group, but the difference was not significant (*p* = 0.062). Interestingly, ERα expression was associated with HPV type. The number of patients with HPV type 16 in the ERα+ subgroup was significantly lower than that in the ER− subgroup (*p* = 0.022). There was no association between ERα expression and sex, age, smoking history, tumor subsite, sample type, surgical margin, and lymphovascular/perineural invasion. *ESR1* mRNA expression was not correlated with any clinicopathologic parameters including HPV type (Table 2).

**Table 2:** Clinicopathologic characteristics with respect to ERα protein and *ESR1* mRNA expression in the p16+/HPV+ OPSCC group

Characteristics	ERα protein (n = 79)			*ESR1* mRNA (n = 71)		
	Positive	Negative	*p* value	Positive	Negative	*p* value
Sex						
Male	33 (91.7%)	36 (83.7%)	0.332	15 (100%)	48 (85.7%)	0.189
Female	3 (8.3%)	7 (16.3%)		0	8 (14.3%)	
Age (years)						
< 65	25 (69.4%)	30 (69.8%)	1	12 (80%)	37 (66.1%)	0.363
≥ 65	11 (30.6%)	13 (30.2%)		3 (20%)	19 (33.9%)	
Smoking history						
Never	13 (36.1%)	17 (39.5%)	0.818	3 (20%)	22 (39.3%)	0.228
Ever	23 (63.9%)	26 (60.5%)		12 (80%)	34 (60.7%)	
Subsite						
Palatine tonsil	31 (86.1%)	37 (86%)	0.589	13 (86.6%)	47 (83.9%)	0.886
Base of tongue	4 (11.1%)	3 (7%)		1 (6.7%)	6 (10.7%)	
Pharyngeal wall	1 (2.8%)	3 (7%)		1 (6.7%)	3 (5.4%)	
Sample type						
Biopsy specimen	13 (36.1%)	12 (27.9%)	0.474	7 (46.7%)	15 (25%)	0.12
Resected specimen	23 (63.9%)	31 (72.1%)		8 (53.3%)	42 (75%)	
Surgical margin^a^						
Clear	19 (82.6%)	20 (64.5%)	0.255	6 (75%)	30 (71.4%)	1
Involved	4 (17.4%)	11 (35.5%)		2 (25%)	12 (28.6%)	
Lymphovascular invasion^a^						
Absent	14 (60.9%)	16 (51.6%)	0.588	5 (62.5%)	21 (50%)	0.704
Present	9 (39.1%)	15 (48.4%)		3 (37.5%)	21 (50%)	
Perineural invasion^a^						
Absent	22 (95.7%)	28 (90.3%)	0.563	8 (100%)	38 (90.5%)	1
Present	1 (4.3%)	3 (9.7%)		0	4 (9.5%)	
Initial stage (8th AJCC)						
I	22 (61.1%)	24 (55.8%)	0.062	7 (46.7%)	34 (60.7%)	0.125
II	14 (38.9%)	13 (30.2%)		8 (53.3%)	16 (28.6%)	
III	0	6 (14%)		0	6 (10.7%)	
HPV type						
Type 16	25 (69.4%)	39 (90.7%)	0.022*	11 (73.3%)	46 (82.1%)	0.475
Type other than 16	11 (30.6%)	4 (9.3%)		4 (26.7%)	10 (17.9%)	
Total	36 (45.6%)	43 (54.4%)		15 (21.1%)	56 (78.9%)	

*ERα* estrogen receptor α, *ESR1* estrogen receptor 1, *HPV* human papillomavirus, *AJCC* American Joint Committee on Cancer

*p < 0.05

^a^Evaluated only in 54 and 50 surgical resection specimens for ERα and ESR1, respectively

### ERα is a favorable prognostic biomarker in both p16+ and HPV+ OPSCC

Next, we performed survival analysis in the cohort of patients with OPSCC (Table 3). Univariate analysis revealed that the p16/HPV status, tumor stage per the 8th edition AJCC system, and ERα and p53 expression are associated with both progression-free survival (PFS) (*p* < 0.001, *p* = 0.004, *p* = 0.044, and *p* = 0.001, respectively) and OS (*p* < 0.001, *p* < 0.001, *p* = 0.002, and *p* = 0.002, respectively). Smoking history was associated only with OS (*p *= 0.037). Multivariate analysis showed that p16/HPV status is an independent and strong prognostic factor in PFS (*p* = 0.001) and OS (*p* = 0.002). Tumor stage and p16/HPV status were found to be co-prognostic factors in OS (*p* = 0.016).

**Table 3:** Univariate and multivariate analysis of the total patient cohort

Characteristics	Progression-free survival			Overall survival		
	Univariate	Multivariate		Univariate	Multivariate	
	*p* value	*p* value	Hazard ratio (95% CI)	*p* value	*p* value	Hazard ratio (95% CI)
Sex	0.925			0.422		
Age	0.746			0.23		
Smoking history	0.265			0.037*	0.41	
Surgical margin^a^	0.472			0.537		
Lymphovascular invasion^a^	0.363			0.96		
Perineural invasion^a^	0.503			0.089		
p16/HPV status	< 0.001*	0.001*		< 0.001*	0.002*	
p16+/HPV− vs. p16+/HPV+		0.028	3.526 (1.142–10.885)		0.003	7.054 (1.912–26.016)
p16− vs. p16+/HPV+		< 0.001	4.334 (1.993–9.427)		0.004	5.616 (1.741–18.121)
8th AJCC stage	0.004*	0.379		< 0.001*	0.016*	
Stage II vs. I					0.151	2.428 (0.724–8.140)
Stage III vs. I					0.001	10.662 (2.508–45.333)
Stage IV vs. I					0.107	3.404 (0.766–15.123)
ERα expression	0.044*	0.406		0.002*	0.107	
*ESR1* mRNA expression	0.116			0.068		
p53 expression	0.001*	0.856		0.002*	0.101	

*HPV* human papillomavirus, *AJCC* American Joint Committee on Cancer, *ERα* estrogen receptor, *ESR1* estrogen receptor 1

*p < 0.05

^a^Evaluated only in 80 surgical resection specimens

**Fig. 3:**
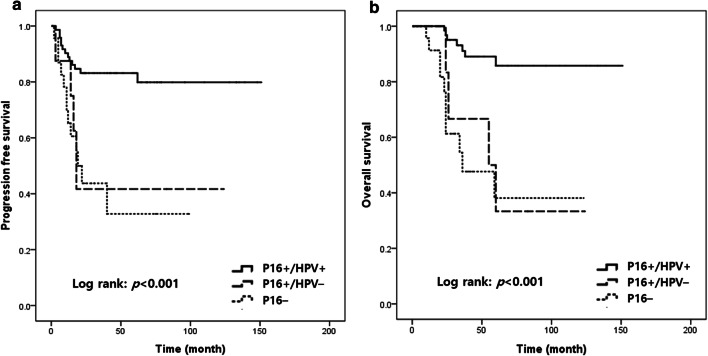
Survival analysis with respect to p16 and human papillomavirus (HPV) status. Kaplan–Meier survival curves for (**a**) progression-free survival and (**b**) overall survival according to p16 and HPV status

**Fig. 4:**
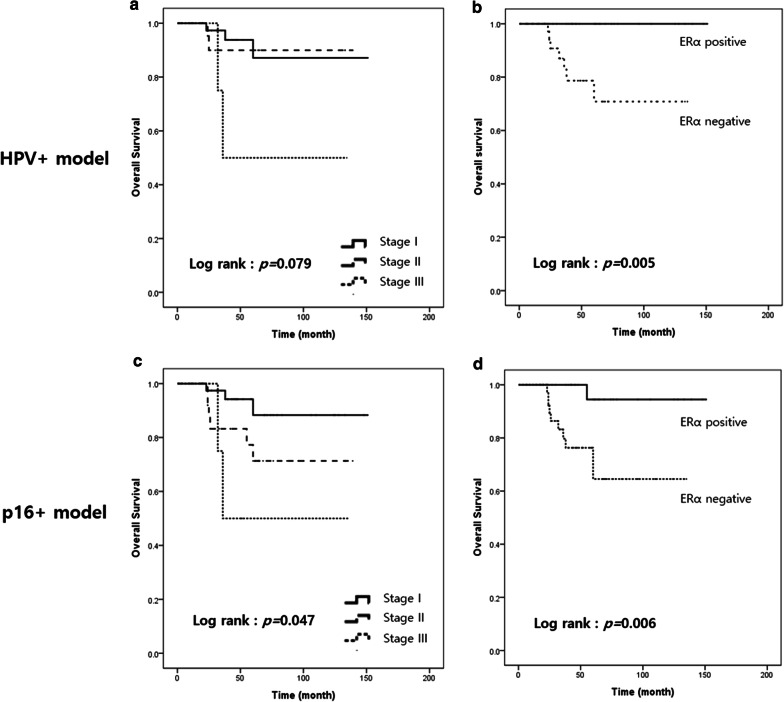
Survival analysis in human papillomavirus-positive (HPV+) and p16+ models. Kaplan–Meier survival curves for overall survival in (**a**, **b**) HPV+ and (**c**, **d**) p16+ models. In the HPV+ model, TNM stage was not associated with survival (**a**), whereas ERα expression was associated with prolonged survival (**b**). In the p16+ model, TNM stage and ERα expression were associated with prolonged survival (**c**, **d**); however, multivariate analysis revealed ERα expression as an independent prognostic factor (*p* = 0.037, hazard ratio: 0.109, 95% confidence interval 0.013–0.871)

Further analysis using Kaplan–Meier curves showed that patients with p16+/HPV− OPSCC showed poor PFS and OS similar to that in patients with p16− OPSCC (*p* < 0.001 for both PFS and OS; Fig. 3a, b). Therefore, we considered the p16+/HPV+ subgroup as the “HPV+ model,” combined the p16+/HPV+ and p16+/HPV− subgroups as the “p16+ model” according to 8th edition AJCC guidelines, and analyzed the prognostic effect of ERα in each model. In the HPV+ model, ERα expression was the only factor that was associated with prolonged OS (*p* = 0.005; Fig. 4a, b). In the p16+ model, ERα and tumor stage were associated with higher OS under the Kaplan–Meier curves (*p* = 0.047 and *p* = 0.006, respectively; Fig. 4c, d). In multivariate analysis, ERα was found to be associated with improved OS adjusted for stage (*p* = 0.037, hazard ratio: 0.109, 95% confidence interval 0.013–0.871) (Table 4).

**Table 4:** Univariate and multivariate analysis of the p16-positive subgroup

Characteristics	Progression-free survival	Overall survival		
	Univariate	Univariate	Multivariate	
	*p* value	*p* value	*p* value	Hazard ratio (95% CI)
Sex	0.923	0.276		
Age	0.805	0.6		
Smoking history	0.519	0.179		
Surgical margin^a^	0.847	0.738		
Lymphovascular invasion^a^	0.793	0.474		
Perineural invasion^a^	0.438	0.061		
HPV type				
Type 16 vs. other than 16	0.061	0.185		
8th AJCC stage	0.333	0.047*	0.234	
Stage II vs. I			0.114	3.071 (0.763–12.358)
Stage III vs. I			0.178	3.471 (0.568–21.196)
ERα expression	0.237	0.006*	0.037*	0.109 (0.014–0.871)
p53 expression	0.192	0.054		

*HPV* human papillomavirus, *AJCC* American Joint Committee on Cancer, *ERα* estrogen receptor

*p < 0.05

^a^Evaluated only in 80 surgical resection specimens

## Discussion

In this study, we investigated the prognostic role of ERα protein expression in patients with OPSCC. We demonstrated that ERα is an independent prognostic biomarker that can complement the 8th edition AJCC staging system in patients with p16+/HPV+ OPSCC and confirmed that p16+ OPSCCs need to be reclassified according to their HPV status.

ERα was expressed in about half of HPV+ OPSCC, unlike the p53 mutation-induced HPV− OPSCC. This is consistent with the previous data from 69 patient samples (44% vs. 17%) [20]. The current values for the frequency of ERα expression in head and neck SCC, including OPSCC, have been variable probably because most previous OPSCCs were HPV−. Although the role of the ERα in OPSCC is not yet clear, it is widely known that ERα plays synergistic roles in cervical carcinogenesis, tumor maintenance, and tumor progression in transgenic mouse models [26–30]. Moreover, aromatase expressed by tumor cells was reported to convert androgen to estrogen and induce the ERα expression in cervical cancer [31]. These findings in cervical cancer indicate the possibility of a similar role of ERα in the pathogenesis of HPV+ OPSCC. However, compared to cervical cancer, OPSCCs exhibit some unique features with respect to ERα expression.

A majority of the basal cells in the normal cervical tissue stained positive for ERα (77–93.7%); however, the frequency of ERα expression in normal oropharyngeal squamous epithelium was lower than that in the cervix (18.7%), and ERα was also expressed in the basal epithelium around the HPV− OPSCC. Koenigs et al. found that ERα is expressed non-uniformly in non-neoplastic tonsil crypt epithelium, and they suggested that this mosaicism could favor ERα-positive normal epithelial cells for HPV infection and genomic integration, leading to OPSCC [19]. However, considering that ERα is expressed in the adjacent basal epithelium of HPV− OPSCC, ERα expression is not limited to the HPV-infected tissue but is likely to generally occur in the oropharyngeal basal epithelium. Furthermore, ERα was expressed only in HPV+ OPSCC and not in HPV− OPSCC, suggesting that ERα influences the development of HPV+ OPSCC by interacting with HPV.

In cervical cancer progression, ERα expression is inhibited in the tumor epithelium but retained in the stromal fibroblasts of the tumor microenvironment [32, 33]. These insights indicate that stromal estrogen signaling and epithelial HPV oncogene expression synergistically promote cervical carcinogenesis. However, in our study, we did not observe ERα expression in the nuclei of stromal fibroblasts of OPSCC. Interestingly, ERα was highly expressed in OPSCC with HPV subtypes (73.3%, 11/15) other than the predominant subtype, HPV type 16 (39.1%, 25/64). On the other hand, *ESR1* mRNA expression did not show a significant difference according to the HPV subtype. Nonogaki et al. suggested that the HPV type 16/18 is responsible for ERα loss in cervical intraepithelial neoplasia and invasive carcinoma of the uterine cervix via post-transcriptional or post-translational regulation [34]. Therefore, we suggest that ERα is involved in the early tumorigenesis stage in HPV+ OPSCC, but in specific HPV type such as type 16, ERα expression may decrease via post-transcriptional regulation, which may be related to tumor aggressiveness.

Although several studies have investigated ERα expression in cervical cancer, the role of ERα as a prognostic factor in cervical cancer remains controversial. Conversely, only a few studies focused on ERα expression in OPSCC but confirmed the association of ERα expression in HPV+ OPSCC with good prognosis [19, 20]. Since the adoption of the 8th edition AJCC, most of the HPV+ OPSCCs were restaged in stage I or II [35, 36], and the patients who received various treatments per the 7th edition AJCC staging system were converged in the same stage per the 8th edition AJCC staging system. Therefore, understanding the applicability of a uniform treatment paradigm in patients with stage I and II HPV+ oropharyngeal cancer has important clinical implications. Although treatment deintensification has been suggested, some patients continued to show poor prognosis, thereby initiating a debate among clinicians about deintensification. In addition, AJCC accepted the classification of HPV+ tumors with p16 IHC only, considering the feasibility of HPV testing [9]. However, recent studies suggested that the application of HPV testing is appropriate for the accurate tumor staging because similar prognoses were reported for p16+/HPV− OPSCCs and HPV− OPSCCs [37, 38]. In our study, compared to the TNM stage, ERα was identified as a better predictor of prolonged OS in patients with p16+/HPV+ OPSCC. Furthermore, ERα was identified as an independent predictor of OS when the TNM stage was adjusted in p16+ model along with the current AJCC recommendation. Therefore, if HPV testing is difficult, performing ERα IHC with p16 may be more helpful for the accurate prediction of clinical outcomes in patients with OPSCC.

Tamoxifen, widely used in the treatment of ERα+ breast cancer, inhibits the expression of the cell cycle- and apoptosis-related genes targeted by ERα [15, 39]; therefore, ERα could be considered as the principal biomarker for response to tamoxifen treatment in HPV+ OPSCC, similar to that in breast cancer. Owing to the availability of these treatment options, hormone therapy could be considered as an adjuvant treatment alternative to chemotherapy or RT because of less adverse effects and reduced risk of recurrence due to deintensification.

Nevertheless, this study has a few limitations. This was a retrospective study that included patients from a single institute; therefore, the number of patients were relatively small, especially that of p16+/HPV− subgroup. Further multicenter and prospective clinical studies are warranted to verify our results and develop an ERα expression-based guideline for deintensification treatment.

## Conclusions

In this study, we demonstrated that ERα is a biomarker for better overall survival in patients with HPV+ OPSCC. Identifying this potential prognostic and therapeutic biomarker may help us improve the patient-specific treatments and develop new deintensification therapies in HPV+ OPSCC.

## Supplementary information

**Additional file 1.** HPV DNA genotyping.

**Additional file 2.** Association between ERα protein and *ESR1* mRNA expression.

## Data Availability

All analyzed and derivative raw data are available on request.

## Abbreviations

AJCC: American Joint Committee on Cancer
CRT: Chemoradiotherapy
ER: Estrogen receptor
ESR1: Estrogen receptor 1
HPV: Human papillomavirus
IHC: Immunohistochemistry
OPSCC: Oropharyngeal squamous cell carcinoma
OS: Overall survival
PFS: Progression-free survival
RT: Radiotherapy
TMA: Tissue microarray
