# Supplementary material for: Estrogen receptor α as a predictive biomarker for survival in human papillomavirus-positive oropharyngeal squamous cell carcinoma
Source: J Transl Med. 2020 Jun 16;18:240. doi: 10.1186/s12967-020-02396-8 (PMC7298756; doi:10.1186/s12967-020-02396-8)
Supplement: Supplementary file 1 — Additional file 1. HPV DNA genotyping. [file 12967_2020_2396_MOESM1_ESM.docx]

**ADDITIONAL FILE 1**

**HPV DNA genotyping**

For all reactions, the reaction mixture volume was 25 µl and contained the template DNA, primer, PNA fluorescence probe [FAM, VIC, ROX, Cy5] set, and Taq DNA Polymerase; all the reagents were available in the kit. Real-time PCR was performed using a QuantStudio 5 Real-time PCR system (Thermo Fisher Scientific, Waltham, MA USA). The following PCR cycle was used: 2-min hold at 50°C and 15-min hold at 95°C for activation; 45 cycles of 95°C for 15 s, 55°C for 45 s, 72°C for 15 s; 95°C for 5 min, 35°C for 5 min, and increment from 35°C to 80°C (0.5°C increment) for melting curve analysis. HPV Mix #A, #B, and #O tubes contain fluorescent dye [FAM, VIC, ROX, or Cy5]- and quencher-conjugated genotype-specific PNA probes for 20 high-risk (16, 18, 26, 31, 33, 35, 39, 45, 51, 52, 53, 56, 58, 59, 66, 68, 69, 70, 73, and 82), 2 low-risk (6 and 11), and 18 low-risk (30, 32, 34, 40, 42, 43, 44, 54, 55, 61, 62, 67, 74, 81, 83, 84, 87, and 90) HPV genotypes, respectively. The HPV genotype was identified by analysing the unique Tm value. PCR efficiency was determined by measuring the threshold cycle (Ct) value of the HPV Mix #O tube Cy5 signal.
