# Supplementary material for: Estrogen receptor α as a predictive biomarker for survival in human papillomavirus-positive oropharyngeal squamous cell carcinoma
Source: J Transl Med. 2020 Jun 16;18:240. doi: 10.1186/s12967-020-02396-8 (PMC7298756; doi:10.1186/s12967-020-02396-8)
Supplement: Supplementary file 2 — Additional file 2. Association between ERα protein and ESR1 mRNA expression. [file 12967_2020_2396_MOESM2_ESM.docx]

**ADDITIONAL FILE 2**

**Association between ERα protein and *ESR1* mRNA expression**

|  | ***ESR1* mRNA expression** | |
| --- | --- | --- |
| **ERα expression** | **Tested sample** | **Positive cases** |
| Negative | 68 | 0 |
| Focal weak to moderate | 11 | 0 |
| Diffuse weak to moderate | 17 | 11 (64.7%) |
| Diffuse strong | 5 | 5 (100%) |
| Total | 101 | 16 (15.8%) |
| Abbreviations: ERα, estrogen receptor α; ESR1, estrogen receptor 1 | | |
